# Model of care for Type 1 diabetes in India: Integrated approach for its incorporation in future national health care policy

**DOI:** 10.1016/j.lansea.2022.05.003

**Published:** 2022-06-09

**Authors:** Masuma Yasmin, Pradip Mukhopadhyay, Sujoy Ghosh

**Affiliations:** Department of Endocrinology and Metabolism, Institute of Post-Graduate Medical Education and Research, Kolkata, 700020, India

Children and adolescents living with Type 1 Diabetes (T1DM) in India face multitude of challenges including lack of free supply of insulin, syringes, glucose measuring devices and strips, lack of structured diabetes education and counselling, and inadequately trained health care professionals.^1^ Multiple daily injections of insulin, self-monitoring of blood glucose, prevention of acute and chronic complications, structured diabetes education, psychosocial support, and safe disposal of sharps are essential components in the management of T1DM.^1^ Absence or disruption of standard care affects the physical and mental well-being of these children.T1DM affects approximately1·2 million children and adolescents worldwide in the age group of 0 – 19 years, with 149,500 new cases being diagnosed every year.^2^ Data from International Diabetes Federation (IDF) Diabetes Atlas 2021 indicates that India has the world's highest number of children and adolescents suffering from T1DM.^2^ But in the absence of a nationwide T1DM registry, it is difficult to estimate exact numbers.

Government of India took initiative to launch the National Programme for Prevention and Control of Cancer, Diabetes, Cardiovascular Diseases and Stroke (NPCDCS) in the year 2008.^3^ This program is adult-centric with no initiatives to address juvenile non-communicable diseases (NCD).^3^ A few non-government organizations and pharmaceutical companies offer some support for T1DM in India.^4^ However, these are all isolated piece meal initiatives. A recent study conducted in a tertiary health care facility in West Bengal, India found that mean glycated hemoglobin (HbA1c) level was 9·1 ± 2·36.^5^ A structured national health care policy is the need of the hour.This could be part of a bigger overarching health care program such as juvenile NCD program.

Extensive literature search revealed that in developing countries, there is no structured health program for T1DM which is implementable, deliverable, replicable, scalable and pharmaco-economically viable.^6^^,^^7^

We have embarked upon implementing a pilot model project for the management of T1DM in selected districts of West Bengal. This is a project-in-innovation, wherein a chronic care model is being developed for providing comprehensive health care for T1DM. The program is being implemented initially in five selected districts of West Bengal. Once the services get established successfully at these health facilities, the program will be scaled up to cover the remaining 23 health districts of West Bengal and later the whole country could possibly benefit from this model if proven to be beneficial and scalable.

The program focuses on providing comprehensive health care services for these children by utilizing the existing health care delivery system and piggy backs on the NPCDCS program. Comprehensive health care services at out-patient department (OPD) basis includingdetection, management of disease and complications, appropriate referral, and rehabilitation, counselling services, building registry of T1DM patients, training human resources, and capacity building at the community level are important components of the program.

Existing NCD clinics of each district hospitalhas been upgraded to T1DMclinic once a week after initial inspection and careful supervision. Existing identified human resources of each district hospital have been trained to provide comprehensive health care for T1DM. Each district hospital has been tagged with a medical college in the state for providing on-site training and hand-holding initially for six months. Children and adolescents with T1DM receive effective treatment at these clinics along with free provision of insulin, glucose measuring devices and strips, routine anthropometric examination, required laboratory investigations, monthly follow up care services, emergency care services and timely referral to tertiary health care facility when necessary.

Structured diabetes education is being provided to the children and their caregivers in the form of counselling services at OPD basis by trained nursing staff, distribution of T1DM education booklets and quarterly educational camps. Children and adolescents with T1DM and their caregivers are trained for self-management, self-monitoring of blood glucose, insulin dose adjustment, sick day rules, nutrition and exercise, and awareness regarding complications. Counselling is the cornerstone for effective management of T1DM because proper self-care ensures good glycemic control and psychological well-being. Children and their caregivers also have access to telephonic contact with trained program coordinator for emergency management. Web-based application has been developed where every treatment detail is recorded and updated in the form of initial, monthly and annual visit formats.

Through this program, long term sustainability of the interventions may be ensuredand mayimprove survival and quality of life of children and adolescents suffering from T1DM, bringing relief to a large section of the community across all classes irrespective of their financial status.We are hopeful that in the long run juvenile NCD program will be launched in India based on our model of care for T1DM.

## Contributors

Conceptualization: Sujoy Ghosh

Writing – original draft: Masuma Yasmin

Writing– review & editing: Pradip Mukhopadhyay

## Declaration of interests

None.
